# Effects on Jump Shooting Accuracy when Using Unstable Surfaces for Functional Balance Training of Youth Basketball Players

**DOI:** 10.5114/jhk/208790

**Published:** 2025-08-03

**Authors:** Nasser Abouzeid Ibrahim, Tomasz Zając, Gabriel Lupu, Mohamed Saad, Miłosz Drozd, Hachim Shani, Alin Larion, Waheed Essa, Thulfiqar Saleh, Mahmoud Hashim, Amr Saber Hamza, Mazin Hasan Alhasany

**Affiliations:** 1 Department of Physical Education, King Faisal University, Al-Ahsa, Saudi Arabia.; 2 Institute of Sport Sciences, The Jerzy Kukuczka Academy of Physical Education in Katowice, Katowice, Poland.; 3 Faculty of Movement, Sports and Health Sciences, Vasile Alecsandri University of Bacau, Bacu, Azerbaijan.; 4 Faculty of Physical Education, Benha University, Benha, Egypt.; 5 Faculty of Physical Education and Sports Science, Almaaqal University, Al Maqal, Egypt.; 6 Faculty of Physical Education and Sport, Ovidius University of Constanta, Romania.; 7 Department of Sports Science and Physical Activity, College of Science, Hafar AL-Batin University, Hafar AL-Batin, Saudi Arabia.; 8 Faculty of Sports Science, Sohag University, Sohag, Egypt.; 9 College of Physical Education, Wasit University, Al Kut, Iraq.

**Keywords:** proprioception, technical skill, postural control, neuromuscular training, lower-limb performance

## Abstract

This study examined the effects of a 10-week functional balance training program using unstable surfaces on dynamic postural control, lower-limb power, and technical shooting performance in adolescent male basketball players. Twenty-one participants were assigned to an experimental group (n = 11; age 16.14 ± 1.13 years) or a control group (n = 10; age 16.89 ± 1.34 years). Both groups trained three times per week over the intervention period. The experimental group completed additional neuromuscular exercises on unstable surfaces, while the control group performed traditional resistance-based routines. Pre- and post-intervention assessments included the Y-Balance Test (YBT), vertical jump height, and jump shooting accuracy (JSA). Statistically significant improvements in dynamic balance (p < 0.001, d = 1.84) and shooting efficiency (p < 0.001, d = 1.78) were observed in the experimental group. The control group also improved significantly in balance (p = 0.021, d = 0.87) and shooting accuracy (p = 0.014, d = 0.95), but to a lesser extent. Between-group comparisons demonstrated significant advantages for the experimental group in both the YBT (p = 0.007, d = 1.00) and JSA (p = 0.004, d = 1.07). No significant improvements were found in vertical jump performance in either group (p > 0.05). These findings suggest that incorporating functional balance training into basketball conditioning programs may enhance postural control and technical shooting execution, with a notable effect size, particularly in dynamic balance. The improvements in Y-Balance scores indicate enhanced sensorimotor control, although further research is needed to clarify the underlying neuromechanical mechanisms.

## Introduction

Basketball performance depends heavily on neuromuscular control and postural stability, particularly during actions that challenge balance, such as jump shots and directional transitions. While dynamic movements characterize gameplay, postural control during the preparatory and execution phases of technical actions remains a critical determinant of success in youth athletes (Montgomery et al., 2010; Zech et al., 2010). In this context, dynamic balance, the ability to maintain stability while the body is in motion serves as a foundational skill underlying coordinated limb movement and skill accuracy.

Functional balance training (FBT), especially using unstable surfaces, is increasingly employed to stimulate proprioceptive pathways and improve neuromuscular efficiency (Hrysomallis, 2011; Zech et al., 2010). Interventions incorporating BOSU balls, balance discs, and similar devices are thought to enhance sensorimotor integration, leading to more stable and controlled execution of motor tasks under varying mechanical demands (Filipa et al., 2010; Myer et al., 2006).

Although the majority of literature emphasizes the role of balance training in injury prevention, emerging evidence suggests that improvements in dynamic balance can facilitate more precise technical execution by improving trunk-limb coordination and reducing compensatory movement patterns (Cressey et al., 2007; Shaffer et al., 2013). In youth athletes, these adaptations may be particularly relevant due to ongoing neuromuscular maturation and the need for efficient interlimb coordination.

Despite this rationale, only few studies have directly examined the impact of functional balance training on basketball-specific skill performance such as shooting accuracy. The literature review indicates that balance training improves technical skills during shooting and dribbling tests (Luo et al., 2023; Wang et al., 2025). It is also worth mentioning that one of the studies indicated no improvement in results during the fast throw, dribbling, defensive slide and lay-up tests (Zacharakis et al., 2020). Therefore, considering the increasing emphasis on practical performance training in children's sports, this gap limits the practical application. The present study sought to address this limitation by investigating whether a 10-week balance training intervention on unstable surfaces could improve dynamic postural control, vertical jump performance, and shooting accuracy among adolescent basketball players. We hypothesized that the experimental group performing neuromuscular balance training would show greater improvements in balance (Y-Balance composite score) and shooting accuracy compared to a control group engaged in traditional strength training. The study aimed to provide evidence-based support for incorporating balance-specific stimuli in youth basketball conditioning programs focused on neuromechanical efficiency and sport- specific skill execution.

## Methods

### Study Design and Participants

This study utilized a quasi-experimental, two-arm pretest-posttest design to evaluate the effectiveness of functional balance training using unstable surfaces on selected neuromotor and performance outcomes in youth basketball players. The primary outcome variables were dynamic balance (Y-Balance Test), lower-limb power (Vertical Jump), and jump shooting accuracy (JSA). These were assessed before and after a 10-week training intervention. The design enabled comparison of within-group improvements over time as well as between-group differences in training-induced changes. No randomization was applied; group allocation was determined by training group affiliation and logistical availability.

The sample size was determined based on an *a priori* power analysis using G*Power 3.1 software. Assuming a medium effect size (f = 0.25), α = 0.05, and statistical power (1–β) of 0.80, the minimum required total sample size was estimated to be 20 participants for repeated measures ANOVA with two groups and two time points. The final sample of 21 athletes (n = 11 EXP, n = 10 CON) thus met the criteria for sufficient statistical sensitivity to detect group-by-time interaction effects across primary outcome variables.

A total of 21 male athletes aged 15 to 18 years were recruited from regional basketball academies. The experimental group (n = 11; mean age = 16.14 ± 1.13 years; body height = 179.29 ± 6.60 cm; body mass = 68.04 ± 5.20 kg; training experience = 6.03 ± 0.9 years) participated in the balance-specific training program. The control group (n = 10; mean age = 16.89 ± 1.34 years; body height = 181.16 ± 5.06 cm; body mass = 72.47 ± 4.20 kg; training experience = 5.00 ± 1.2 years) continued with traditional strength and conditioning practices of comparable frequency and volume. All participants were registered players competing in official youth leagues under national basketball federation regulations, with regular participation in inter-school and club-level competitions.

Pre-intervention testing was conducted one week prior to the start of the training program (late November), and post-testing occurred one week after the completion of the 10-week intervention (early February), during the competitive period of the season.

Participants were eligible for inclusion if they had at least five years of formal basketball training, participated in team practices ≥3 times per week during the in-season period, and had no musculoskeletal injury, orthopedic restriction, or systemic illness in the preceding six months. Athletes were excluded if they had undergone recent surgery, required ongoing rehabilitation, or were using medications that could alter neuromuscular performance. Players were also excluded if they failed to attend more than 80% of the scheduled sessions during the intervention.

This study was designed to test the hypothesis that functional balance training would lead to greater improvements in postural control, power, and shooting performance than traditional resistance training. The experimental protocol, outcome measures, and timepoints were structured to allow for valid statistical comparisons of both intra- and inter-group changes.

### Ethical Considerations

The study protocol was conducted in accordance with and approved by the Bioethics Committee for Scientific Research at the Sohag University, Sohag, Egypt (approval code: 4/II/2023), and met the ethical standards of the Declaration of Helsinki. Written informed consent was obtained from all participants and their legal guardians.

### Training Intervention

**Figure 1:**
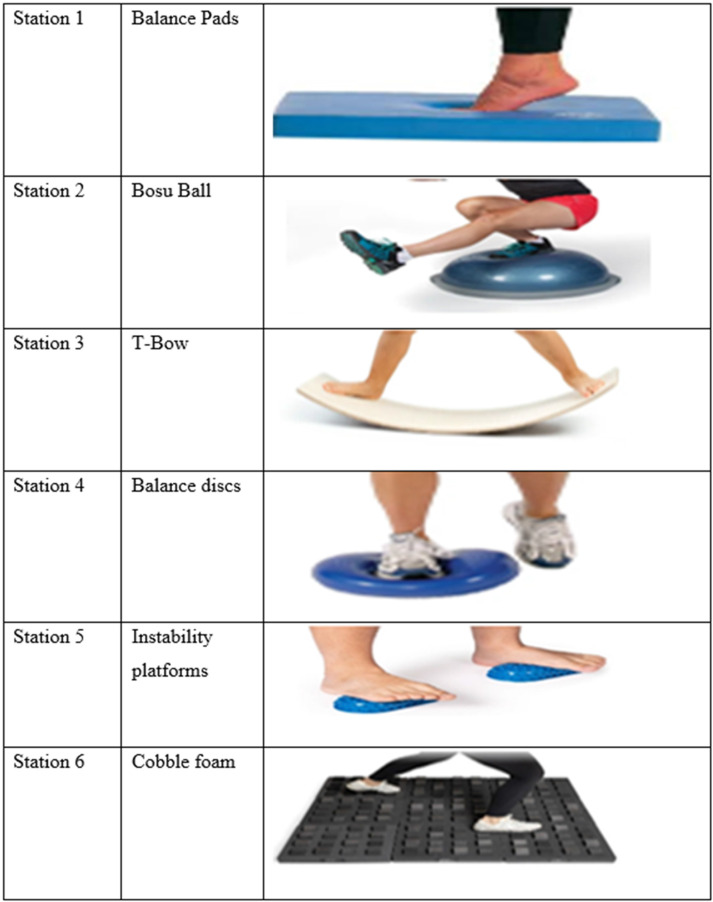
Functional balance training equipment used in the intervention protocol.

The intervention lasted 10 weeks and was conducted concurrently with regular basketball practices. The experimental group performed three sessions per week of functional balance training using unstable surfaces, in addition to their standard team training. Each FBT session consisted of stretching (5 min), exercise circuit (30 min), and a cool-down (5 min). Each session lasted approximately 40 min. Six workstations were organized in a circuit format. The stations involved balance pads, BOSU balls, T-Bows, balance discs, instability platforms, and cobble foam. The components and exercises of the circuit are presented in Figure 1.

Each circuit included task-specific movements such as: (1) a single-leg stance with overhead ball passes while standing on the BOSU, (2) lateral lunges on balance discs, (3) bilateral squats on the T-Bow, (4) unilateral Romanian deadlifts on balance pads, (5) multidirectional perturbation exercises on instability platforms, and (6) dynamic step-to-jump tasks on cobble foam. The goal was to activate core musculature and improve postural reflexes under unstable mechanical conditions.

Exercises were performed using a high-volume, low-intensity interval model (30 s of activity, 60 s of rest, 8–10 repetitions per station). Training progression was monitored by qualified strength and conditioning specialists who provided individualized feedback and ensured proper performance; they increased complexity and neuromuscular demand over time (narrower base of support, multi-directional perturbations, dual-tasking). Progressions were adapted as needed based on the participant’s balance proficiency, ensuring optimal challenge and adherence to the principle of the progressive overload.

The control group followed a conventional resistance training regimen over the same period, matched in volume and frequency but performed on stable surfaces and without balance-specific stimuli.

Each session lasted approximately 40 min and was conducted in-season directly before or after regular basketball practice to optimize training feasibility. The weekly program included three resistance training units focused on multi-joint lower limb strength and core stabilization, with exercises such as squats, lunges, hip thrusts, and Romanian deadlifts. Progression was ensured by increasing the external load (from 50% to 75% 1RM) and introducing instability-free variations of unilateral movements over the 10-week period. All sessions were supervised by qualified strength and conditioning coaches.

To provide additional clarity on the training content, the functional balance training (FBT) protocol implemented in the experimental group is illustrated in Figure 1, which displays the six instability-based stations used during each session. The intervention followed a circuit-based format, with exercises designed to target multi-planar balance control, core stabilization, and reactive postural adjustments.

Each session included six workstations utilizing the following equipment: balance pads, a BOSU ball, a T-Bow, balance discs, instability platforms, and cobble foam. Exercises were selected to progressively challenge neuromuscular coordination and trunk-limb dissociation. The training program evolved bi-weekly by increasing task complexity through narrower bases of support, multi-directional perturbations, and the addition of dual-task components such as cognitive stimuli or sport-specific ball control.

For example, the BOSU ball station featured bilateral squats and single-leg stance with anterior reach; the balance disc station included dynamic lunges with the arm drive; the T-Bow station required lateral hopping and controlled stabilization; the instability platform prompted single-leg Romanian deadlifts with external perturbations; the cobble foam station focused on reactive tasks such as catching and passing; and the balance pad station included plank-based trunk rotations. These exercises are visually referenced in Figure 1.

### Testing Procedures

All performance tests were administered one week prior to and one week following the intervention, under standardized conditions and at the same time of the day. A 15-min dynamic warm-up preceded testing and included jogging, dynamic mobility exercises, and preparatory drills.

### Vertical Jump (VJ)

Vertical jump height was assessed using a standardized wall-marking protocol adapted from Bosco et al. (1983). Participants first established their standing reach height with their dominant hand extended upward while heels remained in contact with the ground. Next, they performed three maximal countermovement jumps (CMJs) with an unrestricted arm swing. The highest point of finger contact on the wall (marked using chalk) was recorded for each trial. Vertical jump height was calculated as the difference between the maximal jump reach and the standing reach height. All measurements were taken with a calibrated wall scale and recorded to the nearest 0.5 cm. The best attempt out of three was retained for statistical analysis.

### Jump Shooting Accuracy (JSA)

**Figure 2:**
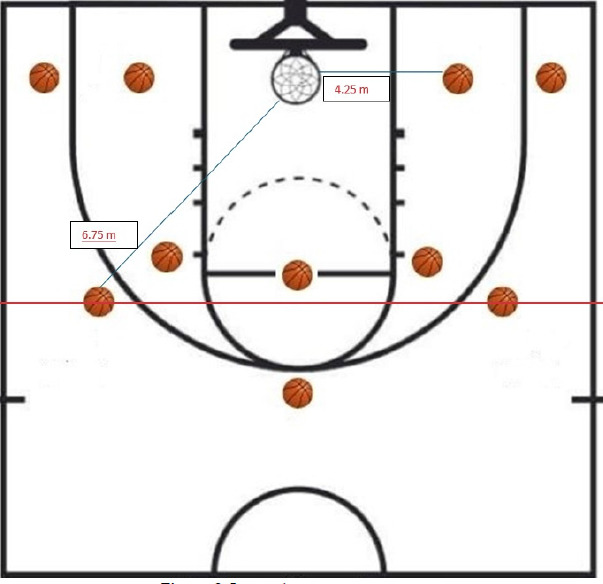
Jump shooting accuracy test.

Jump shooting accuracy was assessed via a standardized test involving 20 jump shots from 10 predetermined zones on the basketball court, as illustrated in Figure 2. Five shooting zones were positioned at a distance of 4.25 m and five at 6.75 m from the basket, covering both 2-point and 3-point attempts. Each shot was scored using a predefined accuracy rubric: 2 points for a made shot, 1 point for the rim contact, 0 for a miss. The total score from each trial was averaged across three rounds to yield the final JSA performance value. Players were instructed to maintain game-intensity pace to simulate real-match shooting contexts.

### Statistical Analysis

All statistical analyses were performed using IBM SPSS Statistics software (IBM Corp. 2021 IBM SPSS Statistics for Windows; Version 27.0) with the level of significance set at *p* < 0.05. The analytical procedures were selected to address both within-group and between-group differences in performance outcomes (Y-Balance composite score, vertical jump height, and jump shooting accuracy) in alignment with the study hypothesis.

Prior to conducting inferential analyses, the assumption of normality was verified for each variable using the Shapiro-Wilk test, and homogeneity of variances was tested with the Levene’s test. All outcome measures met the assumptions required for parametric testing.

Descriptive statistics (mean ± standard deviation) were calculated for each dependent variable at pre- and post-intervention timepoints. To examine whether baseline values differed between the experimental and control groups, independent samples *t*-tests were performed on all outcome variables.

To evaluate training effects within each group, paired samples *t*-tests were used to compare pre- and post-intervention values. Between-group comparisons of pre-post change scores were conducted using independent samples *t*-tests to determine whether improvements in the experimental group significantly exceeded those observed in the control group.

Effect sizes (Cohen’s *d*) were calculated for all significant results to assess the magnitude of the observed differences. Effect size was interpreted according to Cohen’s conventions: small (*d* = 0.2), medium (*d* = 0.5), and large (*d* = 0.8) (Cohen, 1988; Lakens, 2013). In cases where significant improvements were found in both groups, effect sizes were used to contextualize the relative impact of each intervention.

To assess the adequacy of the sample size, a post hoc power analysis was conducted using G*Power software, targeting a medium effect size (f = 0.25), alpha = 0.05, and two-tailed testing. The achieved power was calculated for each primary outcome measure to verify the statistical sensitivity of the design.

## Results

All participants completed the intervention without adverse events. Baseline characteristics of the two groups are presented in Table 1. No statistically significant differences were found between the experimental and control groups at baseline in terms of age (*p* = 0.238), body height (*p* = 0.309), body mass (*p* = 0.074), or training experience (*p* = 0.064).

**Table 1:** Baseline characteristics of the experimental and control Groups (mean ± SD).

Variable	Experimental Group (n = 11)	Control Group (n = 10)	*p*-value
Age (years)	16.14 ± 1.13	16.89 ± 1.34	0.238
Body Height (cm)	179.29 ± 6.60	181.16 ± 5.06	0.309
Body Mass (kg)	68.04 ± 5.20	72.47 ± 4.20	0.074
Training Experience (years)	6.03 ± 0.90	5.00 ± 1.20	0.064

### Y-Balance Test (YB)

In the experimental group, Y-Balance composite scores increased significantly from pre- to post-intervention (86.3 ± 2.4 cm vs. 91.7 ± 3.4 cm), t(10) = 6.12, *p* < 0.001, *d* = 1.84. The control group also showed a significant increase in the dynamic balance test (85.3 ± 2.3 cm vs. 87.3 ± 3.5 cm), t(9) = 2.75, *p* = 0.021, *d* = 0.87. Between-group comparisons showed a statistically significant difference in favor of the experimental group, t(19) = 3.08, *p* = 0.007, 95% CI [1.42, 6.87], *d* = 1.00 (Table 2).

**Table 2:** Pre-Post Comparison of Performance Outcomes and Between-Group Differences.

Variable	Group	Pre-test (Mean ± SD)	Post-test (Mean ± SD)	*p* (within)	Cohen’s *d*	Δ (Mean Change)	*p* (between)	95% CI	*d* (between)
YB (cm)	EXP	86.35 ± 2.41	91.74 ± 3.49	<0.001	1.84	5.39	0.007	[1.42, 6.87]	1.00
	CON	85.33 ± 2.38	87.37 ± 3.51	0.021	0.87	2.04			
VJ (cm)	EXP	45.11 ± 3.15	47.05 ± 3.24	0.092	0.56	1.94	0.372	[−1.37, 3.47]	0.32
	CON	44.92 ± 3.27	46.23 ± 2.87	0.119	0.54	1.31			
JSA (points)	EXP	30.50 ± 2.99	36.00 ± 2.84	<0.001	1.78	5.50	0.004	[1.11, 4.98]	1.07
	CON	30.30 ± 2.87	32.60 ± 3.02	0.014	0.95	2.30			

Abbreviations: EXP: experimental group; CON: control group; YB: Y-Balance test composite score; VJ: vertical jump; JSA: jump shooting accuracy

### Vertical Jump (VJ)

Vertical jump height increased in both groups, however, the changes were not statistically significant. In the experimental group, results increased from 45.1 ± 3.1 cm to 47 ± 3.2 cm, t(10) = 1.87, *p* = 0.092, *d* = 0.56, while in the control group, jump height improved from 44.9 ± 3.2 cm to 46.2 ± 2.8 cm, t(9) = 1.70, *p* = 0.119, d = 0.54. Between-group comparisons revealed no significant difference, t(19) = 0.91, *p* = 0.372, 95% CI [−1.37, 3.47], *d* = 0.32 (Table 2).

### Jump Shooting Accuracy (JSA)

The experimental group showed a significant increase in JSA scores (30.50 ± 2.99 to 36.00 ± 2.84), t (10) = 5.91, *p* < 0.001, *d* = 1.78. The control group also showed an improvement in shooting performance (30.30 ± 2.87 to 32.60 ± 3.02), t (9) = 3.00, *p* = 0.014, *d* = 0.95, yet the between-group comparison demonstrated a significant advantage for the experimental group, t (19) = 3.32, *p* = 0.004, 95% CI [1.11, 4.98], *d* = 1.07 (Table 2).

## Discussion

The current study investigated the effects of a 10-week functional balance training program using unstable surfaces on dynamic balance, vertical jump performance, and jump shooting accuracy in youth basketball players. While both groups demonstrated improvements in balance and shooting accuracy, significantly greater enhancements were observed in the experimental group, particularly in postural control and technical shooting performance. These findings support and extend existing evidence on the role of instability-based training in neuromuscular adaptation, while also emphasizing its novel contribution to skill execution under game-relevant constraints.

Improvements in Y-Balance Test (YBT) performance in the experimental group are consistent with prior studies demonstrating the efficacy of proprioceptive and neuromuscular stimulation via unstable surfaces (Filipa et al., 2010; Zech et al., 2010). Functional instability challenges somatosensory integration, especially during dynamic loading, thereby promoting adaptations in central motor planning and intersegmental coordination (Plisky et al., 2006; Shaffer et al., 2013). Similar effects were described by Marques et al. (2023) who noted enhanced motor control in youth athletes after proprioceptive interventions. In this context, Hamza (2013) argued that balance training should include movement patterns requiring simultaneous force production and stabilization, where one part of the body is dynamically engaged while another maintains control.

The improvements observed in the present study are comparable to those reported in younger or injured populations, suggesting that the applied protocol was effective even in moderately trained adolescents. Hrysomallis (2011) emphasized the essential role of multisensory integration in maintaining balance, while Vom Hofe (1995) suggested that functional balance training facilitated the transfer of strength gains across motor patterns, underscoring its relevance for motor control development in athletic populations. Moreover, Khalili et al. (2022) demonstrated that balance-based interventions could enhance postural stability and reduce symptoms of ankle instability, highlighting the broader neuromuscular benefits of proprioceptive training.

In applied neuromuscular training, the characteristics of balance equipment may influence the specificity and magnitude of adaptation. Research indicates that the mechanical design of tools such as BOSU balls, balance pads, and dynamic platforms elicits differential motor responses (Cressey et al., 2007; Saeterbakken et al., 2014). Devices incorporating multi-directional perturbation such as BOSU balls or air-filled instability platforms enhance proprioceptive activation and trunk stabilization by inducing continuous postural corrections. These findings echo conclusions from a meta-analysis by Li et al. (2025), confirming that unstable surface training improves balance and muscular performance, particularly in youth populations.

Chulvi et al. (2009) highlighted that the widespread adoption of balance-specific tools in sport reflected their effectiveness in targeting both static and dynamic stability. Among these, the T-Bow, which is a curved, multifunctional device, has emerged as a comprehensive training tool capable of simultaneously enhancing strength, flexibility, cardiovascular conditioning, coordination, and balance. Hamza (2013) further noted that wobble boards and similar equipment, operating across multiple planes of motion, allowed for progressive adjustments in difficulty and were suitable for a wide range of athletic abilities. Such tools promote neuromechanical integration by continuously challenging the body’s center of mass relative to a shifting base of support.

These properties may have contributed to the improvements in postural control and skill execution observed in the experimental group. By engaging trunk stabilizers and enhancing sensorimotor coordination under unstable conditions, equipment such as BOSU balls and the T-Bow facilitates the transfer of balance-related adaptations to sport-specific tasks like shooting. These observations align with earlier findings suggesting that multi-modal training approaches can optimize movement efficiency and technical consistency in youth athletes undergoing motor development. In a similar context, Lago-Fuentes et al. (2018) demonstrated that core training on unstable surfaces led to greater improvements in functional fitness compared to training on stable surfaces in professional female futsal players, reinforcing the importance of training surface specificity (Szafraniec et al., 2020).

The lack of significant improvement in vertical jump performance may reflect the principle of training specificity. Unlike plyometric or resistance-based interventions, balance-focused exercises typically involve lower recruitment of high-threshold motor units (Heitkamp et al., 2001; Myer et al., 2006). Although some studies report gains in jump height following instability training in untrained populations (Behm and Colado, 2012; Boccolini et al., 2013; Sakes et al., 2016), such adaptations may plateau in adolescents unless combined with high-intensity resistance stimuli. It is also plausible that concurrent seasonal training or maturational neuromuscular changes influenced minor improvements observed in both groups (Lloyd et al., 2014).

The results regarding jump shooting accuracy are particularly promising. Functional balance training may enhance proximal control and trunk-limb dissociation, thereby enabling more consistent distal mechanics during rapid, high-precision tasks such as shooting. This is consistent with the kinetic chain model, where improved proximal stability contributes to optimized distal output (Cressey et al., 2007; Gabriele et al., 2013; Saeterbakken et al., 2014). Supporting this, Suarez-Balsera et al. (2025) found that trunk stabilization significantly improved accuracy in throwing tasks, highlighting parallels in kinetic sequencing across disciplines. Boccolini et al. (2013) and Barrera-Dominguez et al. (2024) further documented improvements in neuromuscular efficiency and functional performance in basketball following instability training, aligning closely with the findings of the present study.

The novelty of the study lies in its integrated analysis of postural control, power, and sport-specific skills within a single, controlled intervention. While prior literature has primarily focused on injury prevention or general motor capacity, relatively few investigations have linked balance training with basketball-specific technical outcomes (Hrysomallis, 2011; Zech et al., 2010). By including jump shooting accuracy as a primary endpoint, this research contributes applied insights into how neuromechanical adaptation can support sport-specific performance demands.

Several limitations should be considered. The relatively small sample size may have limited the detection of moderate effects, particularly for power-related outcomes. While effect sizes were computed and a post hoc power analysis was conducted, future studies would benefit from larger, randomized samples enabling subgroup and interaction analyses. Additionally, the absence of blinding and randomization might introduce potential bias, despite matched protocols. The reliance on field-based performance assessments, although practical, limits the biomechanical granularity of outcome interpretation. The inclusion of motion analysis, EMG, or kinetic assessments would provide a more detailed understanding of adaptive mechanisms. Furthermore, while test procedures were standardized, the possibility of learning or motivational effects cannot be fully excluded.

Future research should aim to determine the optimal dosage, complexity, and integration strategy of balance training to maximize functional and sport-specific outcomes. Investigations into the combination of instability training with high-load resistance, plyometric, or cognitive dual-task elements may clarify synergistic effects. Biomechanical studies using motion capture or force plates could offer deeper insights into the relationship between core stability and technical execution under realistic game conditions.

In conclusion, the present findings contribute to the growing evidence supporting the integration of functional balance training into performance conditioning for youth basketball players. By linking postural control development with technical skill proficiency, this study offers an applied framework for training strategies that bridge fundamental motor abilities with sport-specific execution.

## Conclusions

This study demonstrates that a structured 10-week functional balance training program using unstable surfaces led to meaningful improvements in dynamic postural control and jump shooting accuracy in youth basketball players. Participants who underwent the balance-focused intervention exhibited greater improvements in balance and technical performance compared to those following conventional resistance-based training. These outcomes indicate that such training may enhance athletes’ ability to stabilize and coordinate body segments during complex sport-specific actions.

Although no significant improvements were observed in vertical jump performance, the observed gains in dynamic balance suggest enhanced sensorimotor integration and functional stability. While the Y-Balance Test does not directly measure neuromechanical control, the improvements in test performance reflect greater dynamic stability, which may support the execution of technical skills under dynamic and unpredictable game conditions.

The findings reinforce the potential value of integrating functional balance training into youth basketball conditioning programs, particularly during phases requiring technical precision and coordination. This approach may contribute to more stable movement patterns and improved performance in tasks that rely on controlled transitions between movement and posture.

## Practical Implications

From an applied perspective, the results support the inclusion of functional balance exercises on unstable surfaces as a complement to traditional strength and skill training in youth basketball. Coaches along with strength and conditioning professionals may consider incorporating balance-focused circuits, using tools such as BOSU balls, balance pads, and T-Bows, particularly during in-season phases where training load management is critical. These exercises appear to enhance neuromuscular control and may facilitate improved execution of technical skills under dynamic and unstable game conditions. Additionally, the improvement in shooting accuracy observed in this study suggests that balance training may indirectly enhance fine motor skill performance by promoting proximal stability and control of kinetic sequencing during ballistic upper-body actions. Importantly, such protocols can be safely implemented in youth populations and require minimal equipment and logistical demands, making them accessible within typical basketball training environments.

## Future Research Directions

Further investigation is warranted to determine the long-term effects and optimal balance training variables for different performance outcomes in basketball. Future studies should aim to include larger, randomized samples and consider stratifying by maturation status to better understand age- and development-related responses. It would be beneficial to compare functional balance interventions across various competitive levels, including elite and professional athletes, to examine whether the observed benefits generalize to more experienced populations. The integration of biomechanical and electromyographic analyses may also offer insight into the neuromechanical mechanisms underpinning the transfer from balance training to technical skill execution. Additionally, research should explore the synergistic effects of combining balance training with strength, plyometric, or dual-task cognitive drills to determine the most effective configurations for enhancing shooting precision, reactive agility, and overall game performance.
